# Impulsive and Compulsive Behaviors in Parkinson’s Disease

**DOI:** 10.3389/fnagi.2014.00318

**Published:** 2014-11-14

**Authors:** Guoxin Zhang, Zhentao Zhang, Ling Liu, Jiaolong Yang, Jinsha Huang, Nian Xiong, Tao Wang

**Affiliations:** ^1^Department of Neurology, Union Hospital, Tongji Medical College, Huazhong University of Science and Technology, Wuhan, China; ^2^Department of Neurology, Renmin Hospital of Wuhan University, Wuhan, China

**Keywords:** Parkinson disease, impulsive control disorders, dopamine dysregulation syndrome, review, dopaminergic replacement therapy

## Abstract

**Background:** Impulsive and compulsive behaviors (ICBs) are a heterogeneous group of conditions that may be caused by long-term dopaminergic replacement therapy (DRT) of Parkinson’s disease (PD). The spectrum of ICBs includes dopamine dysregulation syndrome (DDS), punding, and impulse control disorders (ICDs).

**Contents:** We made a detailed review regarding the epidemiology, pathology, clinical characteristics, risk factors, diagnosis as well as treatment of ICBs.

**Results:** The prevalence of ICBs in PD patients is approximately 3–4% for DDS, 0.34–4.2% for punding, and 6–14% for ICDs, with higher prevalence in Western populations than in Asian. Those who take high dose of levodopa are more prone to have DDS, whereas, ICDs are markedly associated with dopamine agonists. Different subtypes of ICBs share many risk factors such as male gender, higher levodopa equivalent daily dose, younger age at PD onset, history of alcoholism, impulsive, or novelty-seeking personality. The Questionnaire for Impulsive–Compulsive Disorder in Parkinson’s Disease-Rating Scale seems to be a rather efficacious instrument to obtain relevant information from patients and caregivers. Treatment of ICBs is still a great challenge for clinicians. Readjustment of DRT remains the primary method. Atypical antipsychotics, antidepressants, amantadine, and psychosocial interventions are also prescribed in controlling episodes of psychosis caused by compulsive DRT, but attention should be drawn to balance ICBs symptoms and motor disorders. Moreover, deep brain stimulation of the subthalamic nucleus might be a potential method in controlling ICBs.

**Conclusion:** The exact pathophysiological mechanisms of ICBs in PD remains poorly understood. Further researches are needed not only to study the pathogenesis, prevalence, features, and risk factors of ICBs, but to find efficacious therapy for patients with these devastating consequences.

## Introduction

Idiopathic Parkinson’s disease (PD) is a chronic progressive neurodegenerative disorder resulted from dopaminergic cell loss in the substantia nigra (Evans et al., 2005). Impulsive and compulsive behaviors (ICBs), a heterogeneous group of peculiarly additional actions, have emerged as an iatrogenic complication due to dopamine replacement therapy (DRT) in PD patients. ICBs consist of dopamine dysregulation syndrome (DDS), punding, and impulse control disorders (ICDs) (Voon et al., 2006; Mamikonyan et al., 2008; Evans et al., 2009; Wu et al., 2009; Weintraub et al., 2010). DDS occurs mostly due to compulsive overuse of dopaminergic treatment, resulting in secondary cognitive and behavioral disturbances (Evans et al., 2009; Wu et al., 2009). “Punding” is a stereotypical motor behavior characterized by an intense fascination with repetitive, excessive and non-goal oriented handling, and examining of objects (Evans et al., 2004). The main subtypes of reported ICDs include pathological gambling (PG), hypersexuality, compulsive eating, and shopping, of those the first two are more common in male PD patients whereas the last two tend to be more frequently reported in females (Voon et al., 2006; Weintraub et al., 2010). ICDs are considered separately given their proven association with dopamine agonists (DAs) in multiple case control studies (Avanzi et al., 2006; Grosset et al., 2006; Voon et al., 2006; Weintraub et al., 2006), as compared with DDS, which seems to be more closely associated with levodopa (Evans et al., 2005), and punding, whose association with levodopa or DAs is not yet clear (Evans et al., 2004; Lawrence et al., 2007; Miyasaki et al., 2007). ICBs are particularly requisite to recognize because not only these symptoms are under-recognized (Avila et al., 2011a), but they can raise considerable burden to patients, their families, and society. More than a quarter of PD patients with ICBs have two or more behavioral addictions (Ondo and Lai, 2008; Weintraub et al., 2010). In this review, we aim to provide readers with a compressive understanding of the epidemiology, clinical features, pathophysiology, risk factors, and treatment of ICBs, allowing greater insight into potential dominance of ICBs in PD patients.

## Dopamine Dysregulation Syndrome

### Prevalence

Dopamine dysregulation syndrome, also called hedonistic homeostatic dysregulation, is a disturbance that may complicate long-term DRT in PD patients (Evans and Lees, 2004; O’Sullivan et al., 2009). Prevalence of DDS in general population of PD patients is underestimated to be 3–4% (Evans and Lees, 2004; O’Sullivan et al., 2009) as patients are reluctant to discuss their embarrassing behaviors. The available data all come from specialist referral centers with inherent selection biases more or less. In a UK-based study, 15 of 364 PD patients (4.1%) were diagnosed with DDS. Similar results were reported by an Italian movement disorders clinic, with 7 of 202 PD patients (3.4%) exhibiting DDS.

### Characteristic and pathology

Dopamine dysregulation syndrome is characterized by a bizarre phenomenon of an ineffectiveness of medications during “off” periods, and PD patients typically excuse this for maintaining not self-administering doses but an addiction of dopaminergic drugs in excess of those required to control their motor symptoms (Lawrence et al., 2003; Evans et al., 2005). After a period of treatment with high Levodopa equivalent daily dose (LEDD), drug-induced dyskinesias emerge together with socially harmful behaviors (Giovannoni et al., 2000; Merims et al., 2000). Avanzi et al. (2008) reported two male PD patients developing compulsive risk-seeking driving behavior as a result of self-administering high doses of levodopa. Occurrence of DDS was speculated to be due to short duration of levodopa action (Evans and Lees, 2004; Gallagher et al., 2007; O’Sullivan et al., 2009). Recently, DDS was also reported to be related to DA withdrawal syndrome (Limotai et al., 2012). Levodopa is still considered as the most potent trigger of DDS, but subcutaneous apomorphine and oral DAs may also be responsible (O’Sullivan et al., 2009). Additionally, the development of physiological tolerance to levodopa is mediated by central pharmacokinetic and pharmacodynamic changes related to progressive dopaminergic denervation. In PD patients with DDS, however, the psychological effects of “wearing-off” often precede any discernable physical changes. Evidence of maladaptations and sensitization occurring in DDS was noted as enhanced levodopa-induced ventral striatal dopamine release when compared with PD patients who do not compulsively taking dopaminergic drugs (Evans et al., 2006).

### Risk factors and potential mechanisms for DDS development

Personality traits related to impulsivity and sensation seeking (ISS) are associated with drug dependence, drug craving, and vulnerability to relapse. They also play a prominent role in the development of DDS (O’Sullivan et al., 2009). ISS traits, which include novelty-seeking and sensation seeking, are thought to be mediated through mesolimbic dopaminergic systems (Leyton et al., 2002). PD patients with DDS tend to have greater experimental drug use, higher score on ISS ratings, higher alcohol intake and younger PD onset than healthy controls or other PD patients without DDS (Evans et al., 2005; Gallagher et al., 2007; Voon et al., 2007). Besides, disruption of the reciprocal loops between the striatum and structures in the prefrontal cortex following dopamine depletion may predispose to DDS (O’Sullivan et al., 2009). Several studies seeking for candidate genes that may affect the dopamine “D2-like” receptor family and may be associated with impulsive personality traits got conflicting results (Strobel et al., 2003; Tsai et al., 2004). All in all, age at onset and novelty-seeking personality traits are considered to be the two strongest predictors of DDS in PD patients (Evans et al., 2005; Ondo and Lai, 2008).

### Treatment for DDS

Detailed, cautious, and specific enquiries to family members are essential. As outlined in most reports, once PD patients develop DDS, their long-term management becomes difficult (Evans et al., 2005; O’Sullivan et al., 2009; Katzenschlager, 2011). Discontinuation or reduction of dopaminergic drug does, levodopa particularly, should be the first adjustment, which may, however, induce severe motor disorder, depression, or anxiety. Once these conditions occurred, antidepressants should be considered regardless of data available are limited currently about the preferred subtypes of antidepressants in treating DDS. Reports based on cocaine-addicted individuals demonstrated that tricyclic antidepressants might be related with an increased risk of relapse. In addition to pharmaceutical therapies discussed above, deep brain stimulation of the subthalamic nucleus (STN-DBS) is an established therapy for advanced PD patients with the potential not only to allow significant reductions in drug dose but may improve “off”-medication motor symptoms (Weaver et al., 2009). However, the relationship between DDS and STN-DBS remains controversial (Schupbach et al., 2005; Witjas et al., 2005; Bandini et al., 2007; Lim et al., 2009; Eusebio et al., 2013). In 17 PD patients with DDS received bilateral STN-DBS, 12 were resolved or dramatically improved post-operatively (Krack et al., 2003; Funkiewiez et al., 2004; Witjas et al., 2005; Bandini et al., 2007; Knobel et al., 2008), but the remaining 5 patients failed to improve or even worsened (Houeto et al., 2002; Schupbach et al., 2005). Many postoperative PD patients overused dopaminergic drugs to avoid anxiety, dysphoria, and other non-motor symptoms (Evans et al., 2010). The combined effect of DRT and DBS on the limbic territory of the subthalamic nucleus could have precipitated DDS in these patients by inducing hyper-stimulation of the mesolimbic dopamine system (De la Casa-Fages and Grandas, 2012). Continuous jejunal levodopa infusion might have positive effects on DDS, ICDs, and punding as well as motor complications in PD patients (Nyholm et al., 2003, 2005; Odin et al., 2008; Devos, 2009; Catalan et al., 2013). However, the new therapy may not be useful in every instance because it also trigger behavioral abnormalities in sensitive PD patients (Cannas et al., 2013). Due to the putative efficacy of controlling ICBs with levodopa has only been described with small subjects groups (Gerschlager and Bloem, 2009; Valldeoriola and Camara, 2010; Catalan et al., 2013), further randomized controlled trials are urgently needed.

## Punding

### Prevalence

Prevalence of punding varies greatly in different studies because of disparities in study populations, assessment methods (questionnaire or direct interview) as well as diagnostic criteria. It is estimated to be between 0.34 and 14% in PD patients taking high doses of levodopa (> 800 mg/day). However, due to both the absence of related knowledge to punding among clinicians and reluctance of patients to debate their behaviors spontaneously, a widespread under-recognition of punding might have existed up to present (O’Sullivan et al., 2007; Fasano et al., 2008, 2010; Spencer et al., 2011).

### Pathology

Theories on the possible pathophysiology of punding in PD patients are still highly speculative. Previous studies found lesions to the basal ganglia might induce stereotyped behaviors, which resembled punding. Studies supported a causative role of abnormal dopaminergic transmission in the pathogenesis of punding (Spencer et al., 2011). The link between punding and high doses of DRT has already been established (Black and Friedman, 2006; Ferrara and Stacy, 2008; Wolters et al., 2008) including levodopa, DAs, and other alternative medicine (Fernandez and Friedman, 1999; Evans et al., 2004; Miwa et al., 2004; Fasano et al., 2006; Bonvin et al., 2007; Miyasaki et al., 2007). Additionally, the link between punding and dopamine D1, D2 agonists was proved to be closer than D3 and D4 agonists (Fasano and Petrovic, 2010). Moreover, punders were significantly more likely to use dopaminergic drugs acting on D1 receptor such as apomorphine and cabergoline than non-punders (Evans et al., 2004). Suárez et al. (2014) noted that chronic levodopa therapy alters the synaptic efficacy in D1 and D2 striatal medium spiny neurons. However, other researchers failed to find identifiable receptor stimulation profile (Silveira-Moriyama et al., 2006). Recent studies hypothesized that levodopa-induced dyskinesias and behavioral disorders observed in DDS and punding shared common mechanisms (Murer and Moratalla, 2011) involving alterations of glutamate homeostasis with combined activation of sensitized dopamine and NMDA receptors (Kalivas, 2009).

### Clinical characteristics

“Punding” is a set of aimless, stereotypical motor behaviors characterized by an intense fascination with repetitive manipulating and examining familiar objects (Black and Friedman, 2006). Many studies noted punding behaviors often arose from premorbid idiosyncratic habits, occupations, and pastimes (Evans et al., 2004; O’Sullivan et al., 2007). In contrast to other ICBs, punding is not driven by pleasure, anxiety, or obsessions. Patients demonstrate more behaviors that appear similar to traits seen in obsessive–compulsive disorders but more idiosyncratic and less distressing. However, any interruption or disruption of the activity often leads to irritation, anxiety, and frustration (Fullana et al., 2009). Punders are aware of the inapposite obtuse nature of the behaviors but even self-injury resulting from the stereotypes does not abort them (Evans et al., 2004). A positive correlation between the severities of punding and dyskinesia was found (Silveira-Moriyama et al., 2006). Moreover, a 52.8% higher prevalence of dyskinesia in punders over non-punders in Japan was reported (Miwa et al., 2004). Recently, repetitive behaviors are considered as an idiosyncratic side effect to dopaminergic therapy but not a dose- or duration-dependent phenomenon (Nguyen et al., 2010). Independently predictive factors of higher Punding Scale scores include higher impulsivity, poorer disease-related quality of life, younger age of PD onset, and higher daily medication dosage of DAs (Lawrence et al., 2007).

### Treatment

As pathophysiological mechanisms underlying punding have not been elucidated, it is difficult to develop therapeutic methods. The most efficacious strategy is proposed to prevent and diagnose the appearance of punding earlier. Physicians should be aware of normal to abnormal symptoms of punding as these behaviors are frequently undistinguishable and patients can not realize aberrant behaviors spontaneously with impaired insight. Specific treatment strategies should be adopted once punding is diagnosed: (1) the primary method is reduction of dopaminergic medications (Avila et al., 2011b). Symptoms of punding improved strikingly after reduction or cease of levodopa or DAs (Fernandez and Friedman, 1999; Kumar, 2005; Miwa and Kondo, 2008). However, caution is required to get a careful balance between the control of side effects and deterioration of motor symptoms (Aquino et al., 2013). Entacapone could be adopted in case of worsened motor condition (Evans et al., 2004). Selegiline, which was reported to enhance the levodopa function and generate amphetamine-like metabolites, should be avoided in PD patients with punding (Shin, 1997); (2) atypical antipsychotics, such as quetiapine and clozapine, are another therapy that may improve punding symptoms, but curative effect is inconsistent (Fasano et al., 2006, 2011; Bonvin et al., 2007; Miwa and Kondo, 2008; Hardwick et al., 2013). However, atypical antipsychotics are still recommended in controlling episodes of psychosis caused by compulsive DRT use and in raising overnight sleep time (Lawrence et al., 2003); (3) amantadine was recently reported to reduce punding by blocking *N*-methyl-d-aspartic acid receptors (Kashihara and Imamura, 2008; Fasano et al., 2011). However, further evidence to prove the effect is demanded and clinicians should regard the potential induction or worsening of psychosis due to the application of amantadine; (4) whether selective serotonin reuptake inhibitors (SSRIs) is efficacious in punding treatment has come to no conclusion (Kurlan, 2004; Miwa and Kondo, 2008) though SSRIs are shown to be effective in ICDs; (5) data regarding the effect of DBS surgery on punding symptoms in PD patients are limited. Symptoms may worsen, persist, or develop for the first time after DBS regardless of targeting the subthalamic nucleus or the globus pallidus internus (Lim et al., 2009); (6) though low-frequency repetitive transcranial magnetic stimulation (LF-rTMS) has acquired beneficial effects in treating PD and levodopa-induced dyskinesia, effects on punding are uncertain. Recently, Nardone et al. (2013) reported that LF-rTMS produced therapeutic potential for punding, similar to that reported in PD patients with levodopa-induced dyskinesias. However, large-scale, definite studies should still be executed to test the validity of LF-rTMS.

## Impulse Control Disorders

### Prevalence

The frequency of ICDs among PD patients ranges from 6 to 18.4% in Western studies (Fan et al., 2009; Lee et al., 2010; Weintraub et al., 2010; Auyeung et al., 2011; Callesen et al., 2013; Poletti et al., 2013). Weintraub et al. (2010) reported ICDs prevalence of 13.6% in a cross-sectional study by interviewing 3090 idiopathic PD patients in the United States and Canada. Valenca et al. (2013) found a higher ICDs prevalence (18.4%) in Brazil, which might be due to a higher proportion of DAs usage (89.3%). Recently, two studies suggested unexpectedly higher prevalence of 34.8 and 25% in Finnish and French, respectively (Joutsa et al., 2012b; Perez-Lloret et al., 2012). In contrast with Western countries, studies of prevalence and related predisposing factors of ICDs in Asian PD patients are relatively limited (Fan et al., 2009; Auyeung et al., 2011; Lim et al., 2011; Chiang et al., 2012; Tanaka et al., 2013) (Table S1 in Supplementary Material). Auyeung et al. (2011) reported that the prevalence of ICDs was 7% in Chinese PD subjects, which was higher than the previous reported prevalence of 3.53% (Fan et al., 2009). Multiple factors might explain the diverse prevalence of ICDs such as ethnic differences, environmental, cultural, and social factors as well as the dosage and type of dopaminergic medications (Chiang et al., 2012).

### Pathological mechanism

The exact mechanisms for ICDs development remain unknown. Reward-seeking behavior is mediated by the amygdala and nucleus accumbens, both of which receive dopaminergic projections from the ventral tegmental area (Ambroggi et al., 2008). This may be a pathological mechanism underlying DRT and ICDs that matters. Enhanced ventral striatal dopamine release due to hypersensitization of the ventral striatal circuitry may be another explanation (Evans et al., 2006). Complex interactions between DAs and dopamine receptor subtypes may lead to the development of ICDs in PD patients (Pontone et al., 2006). DAs, such as pramipexole and ropinirole, have remarkably specific binding affinities with D3 receptor (Gerlach et al., 2003), which are at least 100-fold greater than with D2 or D1 receptor. As a potential drug target, therefore, D3 receptor known to be localized to the mesolimbic system and involved with reward and motivated behaviors has aroused great concerns (Weintraub, 2008). This might be a momentous explanation why it was not levodopa or carbidopa but DAs that led to ICDs more frequently. Consequently, D3 antagonists might possess therapeutic utility in the resolution of pathological behaviors with elimination or reduction of the D3-preferring agonist (Dodd et al., 2005; Klos et al., 2005; Singh et al., 2007; Bostwick et al., 2009; Hassan et al., 2011). Experiments have been undertaken to test the efficacy of selective D3 receptor antagonists in rat models (Xi et al., 2004; Pak et al., 2006). Except for its larvaceous benefit in animal models (Peng et al., 2009; Khaled et al., 2010; Higley et al., 2011), D3 antagonists have also been administered to humans in single doses for imaging researches (Graff-Guerrero et al., 2010; Searle et al., 2010). CC genotype of the 2B subunit of the glutamate NMDA receptor might be more frequent in PD patients with ICDs than non-affected ones (Lee et al., 2009). Recently, a genetic variant affecting serotonin 2A receptor pathway was found to be associated with ICDs in PD patients receiving DRT, mainly under low-dopaminergic-dose conditions (Lee et al., 2012). Moreover, in comparison with PD patients without ICDs, those who have ICDs present severer cognitive impairments, particularly executive functions (Santangelo et al., 2009; Voon et al., 2010; Barone et al., 2011). Specific difficulties in inhibitory control during cognitive or motor performances have also been involved in the etiology of ICDs development (Seiss and Praamstra, 2004).

### Clinical manifestation

Impulse control disorders are a group of disorders characterized by failure to resist an impulse, drive, or temptation to perform harmful acts to the person or to others. The primary subtypes of ICDs include PG, hypersexuality, compulsive eating, and shopping, of which mere PG is included in the DSM-IV-TR. Except the categories mentioned above, many infrequent behaviors have been reported (Table S2 in Supplementary Material). In recent years, increasing evidence and awareness regarding the relationship between ICDs as motor complications and DRT in PD patients have arisen (Voon and Fox, 2007). The syndrome typically develops in male patients with early onset, orally or subcutaneously administered DRT (Lawrence et al., 2003; Giladi et al., 2007). Comparing 193 PD patients with 190 age/gender-matched healthy controls, Giladi et al. (2007) observed that male gender, younger age at PD motor symptom onset, and longer duration of treatment with DAs contributed independently and additively to the risk of developing ICDs. Except for features discussed, psychiatric co-morbidities are always companied as another important factor predisposing to ICDs.

### ICDs and dopamine agonists

Parkinson’s disease patients treated with DAs are more prone to develop ICDs than those do not use DAs (17.1 vs. 6.9%) (Weintraub et al., 2010). Dopaminergic augmentation of risk-taking behaviors may be a potential contributing mechanism for the emergence of ICDs in PD patients (Claassen et al., 2011). Weintraub et al. (2010) reported that ICD was present in 17.7% of PD patients taking both DAs and levodopa, 14.0% taking DAs, and 7.2% taking levodopa alone. Although the association between dopaminergic therapy and ICDs has been revealed, dose-dependent effect is not yet clear (Weintraub et al., 2010; Ambermoon et al., 2011; Voon et al., 2011a; Joutsa et al., 2012b). Cross-sectional studies are less effective in detecting possible dose-dependent effects because of variations in both the optimal agonist dose for controlling motor symptoms of PD and the severity of ICDs. Longitudinal studies, therefore, prone to be more essential. A few prospective studies investigated the relationship between ICDs and DAs reduction (Singh et al., 2007; Mamikonyan et al., 2008; Macphee et al., 2009; Bharmal et al., 2010; Joutsa et al., 2012a). After following up 290 Finnish PD patients for 15 months, Joutsa et al. (2012a) reported the effect of DAs on ICDs was dose-dependent. Acting on D1 receptor as well as aberrant and excessive expression of D3 receptor in the denervated dorsal striatum (Bordet et al., 1997; Bezard et al., 2003), levodopa tends to be related to ICDs with potential sensitizing or synergistic effects (Weintraub, 2008). Moreover, selegiline, rasagiline (two types of monoamine oxidase inhibitors type B), and lamotrigine (an anti-epileptic drug that decreases glutamate release in the synapse) were also reported to cause ICDs (Grabowska-Grzyb et al., 2006; Shapiro et al., 2006; Reyes et al., 2013).

### Treatment

Management of ICDs in PD patients presents challenges for clinicians because not only patients cannot wake up to aberrant behaviors but also no effective medicines are developed for ICDs. Most evidence reported on management of ICDs comes from empirical data with limited clinical trials to identify efficacious treatment. Once ICDs are diagnosed, patients should reduce or discontinue the dopaminergic drugs carefully to prevent the deterioration of motor symptoms or DA withdrawal symptoms, including anxiety, dysphoria, fatigue, dysautonomia, sleep disturbance, generalized pain, and medication cravings (Rabinak and Nirenberg, 2010). Discontinuing or significantly decreasing DAs exposure, even when offset by an augment of levodopa dosage, is momentous to balance the control of motor disorder and aberrant behaviors (Dodd et al., 2005; Mamikonyan et al., 2008). Besides, cognitive-behavioral therapies are receiving empirical support in non-PD populations and may be encouraged in PD patients (Carroll and Onken, 2005; Dowling et al., 2006). Moreover, antipsychotic drugs such as olanzapine or quetiapine coupled with either DAs or levodopa may be efficacious in ICDs treatment (Grant et al., 2008). The relationship between ICDs and DBS was reported with inconsistent results. PG improved or disappeared after bilateral STN-DBS in nine patients (Molina et al., 2000; Ardouin et al., 2006). However, in the remaining 18 patients (11 bilateral, 1 unilateral, and 6 not specified), ICDs or punding developed or turned decompensated post-operatively (8 PG, 7 hypersexuality, 2 punding, 1 intermittent explosive disorder, and kleptomania) (Romito et al., 2002; Kleiner-Fisman et al., 2003; Sensi et al., 2004; Machado et al., 2005; Ardouin et al., 2006; Lu et al., 2006; Morgan et al., 2006; Smeding et al., 2006, 2007). Selective stimulation of limbic region of STN and surrounding structures was also reported to trigger or worsen non-motor side effects (Voon et al., 2005, 2008; Frank et al., 2007; Mallet et al., 2007; Alberts et al., 2008; Moum et al., 2012). Therefore, many reports speculated the positive outcomes after STN-DBS might be related to discontinuation of dopaminergic treatment after surgery (Santangelo et al., 2013). Castrioto et al. concluded that STN-DBS could not only improve motor and non-motor behaviors, but induce excessive motor, cognitive, and emotional behavioral disinhibition. Fine-tuning of stimulation parameters with dopaminergic drugs was necessary to prevent or improve pathological behaviors (Castrioto et al., 2014).

## Risk Factors for the Development of ICBs

Multiple risk factors contributed to the occurrence of ICBs. The association between dopaminergic drugs treatment, particularly DAs in higher dosages, and ICBs has raised much concerns (Lawrence et al., 2003, 2007; Maia et al., 2003; Evans and Lees, 2004; Klos et al., 2005; Pontone et al., 2006; Giladi et al., 2007; Voon and Fox, 2007; Voon et al., 2007; Mamikonyan et al., 2008; Weintraub et al., 2010; Auyeung et al., 2011). Giladi et al. (2007) demonstrated it was not only the exposure to DAs that was associated with the development of addiction-like behaviors but the behaviors were dose- and time-dependent. Many other potential risk factors have also drawn much attention. Male gender, younger age at PD onset, levodopa treatment (especially when given in combination), higher novelty-seeking personality traits, and psychosocial factors such as substance abuse and depression may also predispose patients to developing ICBs (Lawrence et al., 2003, 2007; Evans and Lees, 2004; Evans et al., 2004, 2005, 2009; Voon et al., 2006, 2007; Giladi et al., 2007; Wu et al., 2009; Poletti and Bonuccelli, 2012). Pezzella et al. screened 202 PD patients for DDS and found a significant correlation between DDS and both history of mood disorders and previous use of DRT, especially DAs, either as monotherapy or in combination. Joutsa et al. (2012a) concluded that ICDs were not only more frequent in men but also approximate six times more unlikely to be resolved compared to women after a 15-months follow-up. Sossi et al. (2006) pointed out that younger sick brain was likely to be more sensitive to DRT, and dopamine turnover in younger-onset patients undergone a greater alteration and thus likely led to a striking imbalance between dopamine synthesis, storage, and release. Moreover, greater impulsive choice, faster reaction time, faster decision conflicting reaction time, and executive dysfunction might also contribute to ICDs in PD patients (Voon et al., 2010). Lately, Voon et al. (2011b) proposed that ICDs are associated with multiple psychiatric and cognitive impairments such as affective and anxiety symptoms, elevated obsessionality, novelty-seeking, and impulsivity. The relation between PG and depressed mood were also observed (Potenza et al., 2005; Grant and Potenza, 2006; Romer Thomsen et al., 2009). Major depression in middle-aged men appeared to be frequently comorbid with PG, with over-lapping genetic factors contributing substantially to the co-occurrence (Potenza et al., 2005; Romer Thomsen et al., 2009). The exact effect of STN-DBS on addictive behaviors in PD patients remains to be further investigated. The postoperative motor improvement resulting from STN-DBS showed the potential to allow significant reductions in drug dose (Schupbach et al., 2005; Witjas et al., 2005; Bandini et al., 2007; Lim et al., 2009). However, others found no obvious improvement or even worsened symptoms after surgery (De la Casa-Fages and Grandas, 2012).

## Assessment of Impulsive–Compulsive Behaviors in Parkinson’s Disease

Assessment instruments for ICBs and related disorders in PD patients are limited. Diagnostic criteria are defined in the DSM-IV-TR. Though consisted of many questions about compulsive gambling, buying, and sexual behavior, the Minnesota Impulsive Disorders Interview does not match the style of DSM-IV-TR and assesses inadequate contents of ICDs. Other disorder-specific screening instruments used in ICBs including the South Oaks Gambling Screen, the Buying Questionnaire, and the punding questionnaire, are also limited with no existing single instrument fulfilling the criteria of being comprehensive, self-rated, and validated for use. Though the DDS-Patient and Caregiver inventory (Cabrini et al., 2009) and the Movement Disorder Society-Unified PD-Rating Scale (Goetz et al., 2008) were developed lately to assess the presence of a range of impulsive–compulsive behaviors in PD patients, their validity as assessment tools remains to be further proved (Goetz et al., 2012). The Questionnaire for Impulsive–Compulsive Disorders in Parkinson’s Disease (QUIP) was recently developed and validated to detect the presence of clinically significant ICDs, DDS, and other compulsive behaviors (punding, hobbyism, and walkabout) (Weintraub et al., 2009). The QUIP is consisted of three sections: Section 1 assesses four ICDs (involving gambling, sexual, buying, and eating behaviors); Section 2 tests other compulsive behaviors (punding, hobbyism, and walkabout); and Section 3 checks compulsive medication use (Weintraub et al., 2009). Recently, QUIP was used to screen the prevalence of ICBs and subsyndromal ICBs of PD patients in Malaysia (Lim et al., 2011) and German (Rohde et al., 2013). Papay et al. (2011) assessed observer variability and validity in reporting ICD symptoms using the QUIP. Besides, Tanaka et al. (2013) developed a Japanese version of QUIP (J-QUIP) and evaluated the sensitivity, specificity, positive predictive value, and negative predictive value for each behavior via diagnostic concordance rate between actual diagnosis and result of J-QUIP in Japanese PD patients. However, the QUIP is designed to be sensitive but not highly specific (i.e., it over identifies patients) for ICBs detection, so a positive result should be followed by a comprehensive clinical screen to determine the validity and severity of symptoms, as well as need for clinical management (Weintraub et al., 2009). Moreover, the Questionnaire for Impulsive–Compulsive Disorder in Parkinson’s Disease-Rating Scale (QUIP-RS), based on the QUIP, was developed. In contrast to its predecessor, QUIP-RS requires individuals to rate the severity of each symptom based on its frequency using a five-point Likert scale. The QUIP-RS detects subsyndromal behaviors and establishes clear cut-off points with a good balance between sensitivity and specificity (Weintraub et al., 2012).

## Conclusion and Future Prospection

**Figure 1:**
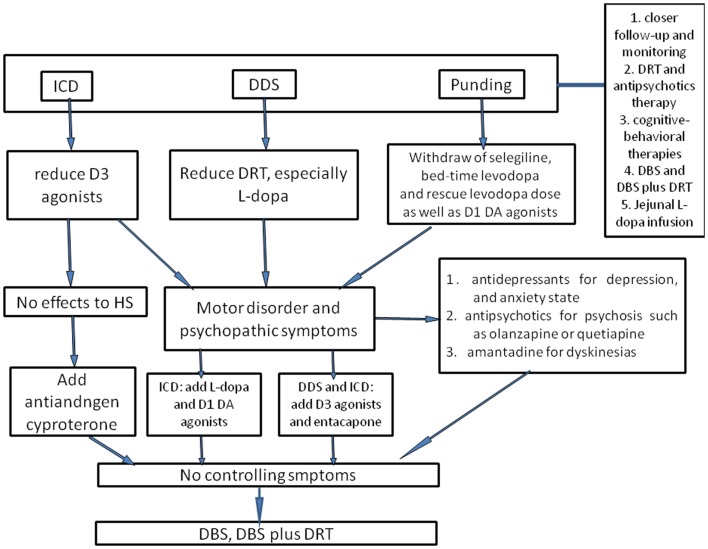
**The flowchart of management of ICBs in Parkinson’s disease**. To prevent ICBs occurring, closer follow-up and monitoring are essential. Once ICBs are diagnosed, the first-choice is adjustment of dose of DAs and levodopa, but this requires a good balance between ICBs symptoms and motor disorders. For ICDs, D3 agonists should be reduced; for DDS, the dose of levodopa is considered to be cut down. With regard to punding, selegiline, which enhances the levodopa action and has amphetamine-like metabolites, and bedtime DRT should be withdrawn as well as reducing levodopa dose. In case motor disorder and psychopathic symptoms occur, levodopa may be useful for ICDs, and D3 agonists or entacapone may alleviate DDS and punding as well as antidepressants, antipsychotics, and amantadine. However, one should be ever vigilant that these could trigger or worsen concomitant disorders. Antiandrogen cyproterone should be considered for hypersexuality, especially when there are no other effective drugs. Atypical neuroleptic drugs could also be used for ICBs, such as olanzapine or quetiapine, especially for punding patients presenting with psychosis or reduced sleep time. Besides, cognitive-behavioral therapies have been evaluated as an efficacious method. Supposing that all interventions discussed above fail to control symptoms, DBS or DBS plus DRT could also be considered. Moreover, jejunal levodopa infusion was recently found to be effective for ICBs, whereas the availability was merely evaluated in small sample, and thus further larger clinical studies are still needed.

Though mounting data regarding ICBs have aroused great concerns in recent years, ICBs remain a great challenge in clinical practice (Figure 1). Vigilance in the prescribing physical symptom is of paramount importance. Exact prevalence rate of ICBs is still frequently underreported. As PD patients with ICBs have reduced insights into social consequences of their behaviors, it turns momentous to screen ICBs using specifically devised and validated diagnostic tools and to enquire relevant information from both patients and caregivers. Susceptibilities to ICBs in PD patients have been associated with specific demographic and clinical characteristics and abnormalities on functional neuroimaging studies including male gender, higher LEDD, young age at PD onset, personal or family history of alcoholism, and impulsive or novelty-seeking personalities. The pathology of PD, including the role of different neurotransmitters, likely plays a facilitative role in developing ICBs but the exact mechanisms remain to be established. Numerous studies have related ICBs to dopaminergic therapy. Imaging studies also provided additional evidence to strengthen the link. Given the potentially devastating consequences and lack of effective treatments for ICBs, preventive strategies do matter. Though ICBs have been considered as side effects resulting from DRT, levodopa remains the gold standard for symptomatic treatment in PD patients and should not be withheld from patients especially in whom sufficient symptomatic control cannot be otherwise obtained. Whether or not a DA should be used as early monotherapy largely depends on the perceived risk of dyskinesias – for which younger age is a major determinant (Antonini et al., 2009). Readjustment of DRT dose is still the main way to prevent and control ICBs, but attention should be drawn to balance ICBs symptoms and motor disorders. Moreover, atypical antipsychotics, antidepressants, amantadine, and psychosocial interventions can also be prescribed in controlling episodes of psychosis caused by compulsive DRT use with cautiousness because these drugs may also induce variable degree of motor function worsening. In addition to the pharmacal therapies described above, STN-DBS may be another efficacious therapy for advanced PD patients with the potential not only to allow significant reductions in drug dose but to improve “off”-medication motor symptoms (Table S3 in Supplementary Material). Furthermore, Williams et al. (2010) reported that surgery plus best medical therapy improved patient self-reported quality of life more than best medical therapy alone in patients with advanced PD. All in all, the use of emerging, empirically validated treatments for ICBs should be considered, particularly for patients whose discontinuation of DA therapy is not a viable option. Despite many achievements have been acquired, several unanswered questions remain. Firstly, what are the main reasons that differentiate PD patients that develop ICBs from those patients do not develop ICBs even if they receive the same medication? Although we have found many risk factors for the development of ICBs, this question is still unanswered at the level of neuronal function. Furthermore, why do different PD patients develop differing ICBs presentations? Individual susceptibilities, disease process, and dopaminergic medications might all contribute to the occurrence of diverse ICBs, but the accurate nosogenesis should be further investigated. Finally, what is the relationship between ICDs, DDS, punding, and substance abuse? Though familiar clinical risk factors and some common grounds have been observed, the key factors leading PD patients to substance abuse but not ICDs, DDS, or punding has not been illustrated. In conclusion, the current goal is to determine the exact mechanisms in the pathogenesis of ICBs in PD patients, and then find the corresponding therapy to offer robust motor benefit without producing ICDs or other serious side effects.

## Author Contributions

Tao Wang and Zhentao Zhang contributed to the conception, Guoxin Zhang and Zhentao Zhang participated in designing the study, acquired data, and drafted the manuscript; Jinsha Huang, Ling Liu, and Jiaolong Yang were involved in collecting data; and Guoxin Zhang, Zhentao Zhang, and Nian Xiong reviewed and edited the manuscript. All authors have read, revised, and approved the final manuscript.

## Conflict of Interest Statement

The authors declare that the research was conducted in the absence of any commercial or financial relationships that could be construed as a potential conflict of interest.

## Supplementary Material

The Supplementary Material for this article can be found online at http://www.frontiersin.org/Journal/10.3389/fnagi.2014.00318/abstract

## Abbreviations

DAs, dopamine agonists; DDS, dopamine dysregulation syndrome; DRT, dopamine replacement therapy; ICBs, impulsive and compulsive behaviors; ICDs, impulse control disorders; ISS, impulsivity and sensation seeking; LEDD, levodopa equivalent daily dose; LF-rTMS, low-frequency repetitive transcranial magnetic stimulation; NMDA, *N*-methyl-d-aspartic acid; PD, Parkinson’s disease; PG, pathological gambling; QUIP, questionnaire for impulsive–compulsive disorders in Parkinson’s disease; SSRIs, selective serotonin reuptake inhibitors; STN-DBS, deep brain stimulation of the subthalamic nucleus.
