# Supplementary material for: Impulsive and Compulsive Behaviors in Parkinson’s Disease
Source: Front Aging Neurosci. 2014 Nov 14;6:318. doi: 10.3389/fnagi.2014.00318 (PMC4231987; doi:10.3389/fnagi.2014.00318)
Supplement: Supplementary file 1 [file Table_1.DOC]

| **First author** | **Area** | **Participants** | **Measures** | **Prevalence** | **Conclusions** |
| --- | --- | --- | --- | --- | --- |
| **Auyeung et al.** | Hong Kong | 213 | Structured screening questionnaire | ICD:7% | Dose of DA, young age at PD onset, a history of anxiety or depression were independent predictors for developing ICD. |
| **Lim et al.** | Malaysia | 200 | QUIP | ICD:15.4%  DDS:2.0%  Punding:13.8% | Asian patients with Parkinson’s disease may be susceptible to dopaminergic medication-related side effects |
| **Chiang et al.** | Taiwan | 268 | interview | ICD:4.85%  DDS:1.12%  Punding:0.37% | Prevalence of ICB is lower in Taiwan compared with the Caucasians. possible reasons including diﬀerences in ethnicity, environmental, cultural, and social factors as well as the dosage and selection of dopaminergic medications. |
| **Tanaka et al.** | Japan | 118 | Japanese version of QUIP | ICD:12.9%  DDS:2.2%  Punding:6.5% | J-QUIP as a screening questionnaire for ICBs is available with similar prevalence of ICBs to Western countries. |
| **Fan et al.** | Chinese Mainland | 400 | modiﬁed  SOGS, two Structured screening questionnaires and telephone interview | ICD:3.21%  DDS:0.64% | PD patients who took dopamine agonists were more likely to report ICD behaviors. |
| **Lee et al.** | Korea | 1167 | a modiﬁed version of the Minnesota  Impulsive Disorders Interview | ICD:10.1%  Punding:4.2% | The dose of dopaminergic medication is signiﬁcantly associated with the development of  ICBs, except compulsive eating in PD. |

**Table 1: Prevalence and predisposing factors of ICDs in Asian PD patients.**

QUIP: the Questionnaire for Impulsive-Compulsive Disorders in Parkinson’s Disease; J-QUIP : Japanese version of QUIP; SOGS: the South Oaks Gambling Screen.
