# Supplementary material for: Impulsive and Compulsive Behaviors in Parkinson’s Disease
Source: Front Aging Neurosci. 2014 Nov 14;6:318. doi: 10.3389/fnagi.2014.00318 (PMC4231987; doi:10.3389/fnagi.2014.00318)
Supplement: Supplementary file 2 [file Table_2.DOC]

| **First author** | **Gender** | **Presentation** | **New behaviors** | **Speculated reasons** |
| --- | --- | --- | --- | --- |
| **Hassan et al.** | M:7  F:4 | Compulsive hobbying (Flower arranging, Fishing etc) | Y | DAs |
| **Bienfait et al.** | M | Impulsive smoking and grooming beard | Y | DAs |
| **McKeon et al.** | M: 4  F: 2 | weighing, video game playing, fishing, gardening, locking and unlocking doors, repetitive dressing and undressing | U | DAs |
| **Machado et al.** | F | Trichotillomania (TTM) | N | STN-DBS |
| **Bonfanti et al.** | F | Kleptomania | Y | DAs and CL |
| **Sensi et al.** | M | kleptomania | Y | STN-DBS |
| **Wu et al.** | M: 25  F: 4 | Problematic Internet use | U | U |

**Table 2:** **Unfrequent behaviors reported in ICDs.**

M: male; F: female; Y: yes; U: unknown; CL: Carbidopa/levodopa; DAs: dopamine agonists; STN-DBS: subthalamic nucleus deep brain stimulation.
