# Supplementary material for: Impulsive and Compulsive Behaviors in Parkinson’s Disease
Source: Front Aging Neurosci. 2014 Nov 14;6:318. doi: 10.3389/fnagi.2014.00318 (PMC4231987; doi:10.3389/fnagi.2014.00318)
Supplement: Supplementary file 3 [file Table_3.DOC]

**Table 3: Efficacy of DBS in treatment of PD patients with ICDs, DDS or punding.** DBS: deep brain stimulation; M: male; F: female; LEDD: Levodopa equivalent daily dose; U: unknown; N: no; Y: yes; DDS: dopamine dysregulation syndrome; PG: pathological gambling; HS: hypersexuality; ICB: impulsive and compulsive behaviors; ICD: impulse control disorders; BE: binge eating; CS: compulsive shopping; ES: excessive shopping; Levo: Levodopa; Pra: Pramipexole; Bro: Bromocriptine; Per: pergolide; AI: apomorphine injection; Car: carbidopa; Ent: entacapon; Pro: Propranolol; Bi: Biperiden; Am: Amantadine; Se: Selegiline.

| Reference | Patients | Gender | Age of  onset PD | Age at  DBS surgery（Y） | LEDD-  Before  (mg/day) | LEDD-  After  (mg/day) | Psychiatric  illness | ICB types before DBS | ICB types after DBS | STN-DBS |
| --- | --- | --- | --- | --- | --- | --- | --- | --- | --- | --- |
| Moum et al. | 159 | M:113  F:46 | 48.54 | 61.39 | 888.8 | U | U | ICD:7  DDS:5 | ICD:16  DDS:7 | unilateral |
| Sensi et al. | 1 | M | 56 | 64 | 1000 | 700 | N | N | aggressive behavior disorder | bilateral |
| Bandini et al. | 2 | M | 39 | 43 | Levo: 800  Pra: 4.2 | Levo: reduced  Pra: discontinued | N | PG | N | bilateral |
|  |  | M | 46 | 51 | Levo:1200  Bro: 30  Par: 60 | Levo: 200 | U | PG/DDS | N | bilateral |
| Smeding et al. | 1 | M | 53 | 63 | Levo: 600  Per: 6-8 | Levo:400  Per:3 | N | N | PG | bilateral |
| Machado et al. | 1 | F | U | 58 | U | U | Y | Trichotillomania | wound complications | bilateral |
| Ardouin et al. | 7 | M:6  F:1 | U | 54 | 1395 | 571 | Y | PG :7  DDS: 4 | N |  |
| Ardouin et al. | 1 | M | 37 | 59 | Levo:1000  Bro: 45  AI: 3.5 | levo: 225  Bro:15 | N | PG | apathy | bilateral |
| Witjas  et al. | 2 | M  M | 30  48 | 38  53 | 2500  1450 | 0  300 | N  N | DDS,HS  DDS | N  N | bilateral |
| Morgan et al. | 1 | M | 52 | 59 | U | U | N | N | Self-Stimulatory Behavior | unilateral |
| Knobel et al. | 1 | m | 39 | 55 | 1830 | 560 | Y | DDS, PG, HS | N | U |
| Pallanti et al. | 5 | M:3  F:2 | U | 63.8 | 1225 | 954.25 | N | N | Punding:5;  HS:1; PG:1 | unilateral |
| Eusebio et al. | 18 (DA misuse:) | M:16 | 42.7 | 52.5 | 1466 | 738.1 | Y | DDS:12; PG: 9; ES: 7;  HS: 13; BE: 3; Punding:3; | DDS:1; HS:1;  BE:5;  Punding: Improved | bilateral |
| Eusebio et al. | 92 (No DA misuse) | M:65 | 50.5 | 61.9 | 1169 | 697.5 | N | DDS: 0; PG:5; ES:7;  HS:12; BE:7; Punding:7; | DDS:0; PG:1;  ES:2; HS:1;  BE:21;  Punding: Improved | bilateral |
| Lim  et al. | 6 | M:3  F: 3 | U | U | U | 329 | Y | DDS:6; Punding: 4; PG: 2; HS: 4; CS: 3. | None: 3;  improved: 3 | bilateral |
| Lim  et al. | 11 | M:10  F:1 | U | U | U | 2250 | Y | DDS:8; HS:2; PG:3;  Punding:6, CS:1 | No improvement: 6;  worsened: 2;  new DDS: 2;  de novo PG: 1 | bilateral |
| Halbig  et al. | 3 | U | 52(m) | 64(m) | 1338 (M) | 467 (M) | U | N | CS: 2; PG: 1 | U |
| De la Casa et al. | 3 | F | 37 | 47 | 750 | 1000 | N | N | DDS; BE | bilateral |
|  |  | M | 52 | 59 | 1500 | U | Y | N | DDS | bilateral |
|  |  | M | 30 | 45 | Levo: 600; Car: 150; Ent: 800; Pro:160; Bi: 4; Am: 100; Se: 5  Pra: 2.8 | U | N | N | DDS;  HS;  apathy | bilateral |
